# Determining Water Transport Kinetics in Limestone by Dual-Wavelength Cavity Ring-Down Spectroscopy

**DOI:** 10.1021/acs.analchem.1c04277

**Published:** 2022-02-08

**Authors:** Dáire E. Browne, Robert Peverall, Grant A. D. Ritchie, Heather A. Viles

**Affiliations:** † Department of Chemistry, Physical and Theoretical Chemistry Laboratory, University of Oxford, South Parks Road, Oxford OX1 3QZ, United Kingdom; ‡ School of Geography and the Environment, University of Oxford, South Parks Road, Oxford OX1 3QY, United Kingdom

## Abstract

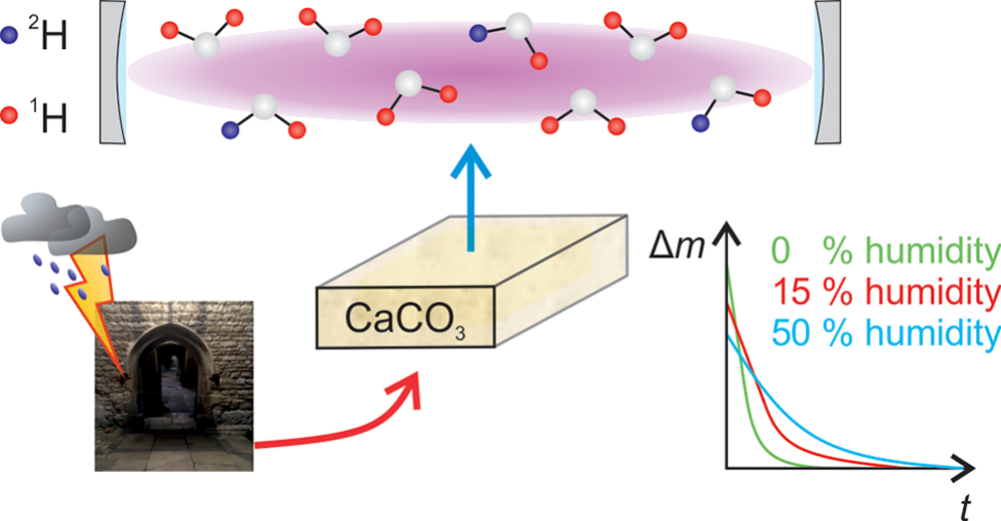

Water plays a major role in the deterioration of porous building materials such as those widely found in built heritage, influencing many physical, chemical, and biological decay processes. This article details a proof-of-principle study using near-infrared cavity ring-down spectroscopy (CRDS) to monitor the release of water and its artificially enriched isotopologues from small (ca. 25 × 25 × 5 mm) samples of limestone subject to drying by a fixed flow of nitrogen with varying levels of humidity and at room temperature and atmospheric pressure. Under low-humidity conditions, the drying kinetics are consistent with the well-established two-phase drying process exhibited by porous materials, namely, an initial constant drying rate period (phase I) followed by a falling drying rate period (phase II). The water diffusivity during phase II, *D*_II_, was measured (for Clipsham limestone) to be 3.0 × 10^–9^ ± 1 × 10^–10^ m^2^ s^–1^. The CRDS measurements allow spectroscopic determination of the total mass of water released by the sample, and the calculated values are in excellent agreement with gravimetric analysis. Importantly, the selectivity and sensitivity afforded by CRDS allows isotope analysis to be carried out, such that the flux of isotopically labeled water out of the sample can be determined under conditions of humidified flow where there may be a simultaneous ingress of water from the environment. Dual-wavelength CRDS distinguishes isotopic species, and it is demonstrated that the drying kinetics and physical properties of the samples are self-consistent when monitoring both HDO and H_2_O (for HDO, *D*_II_ was 3.2 × 10^–9^ ± 4 × 10^–10^ m^2^ s^–1^). As the humidity levels in the flow increase, a departure from the distinct two-phase behavior is observed in the HDO drying curves. These new measurements of isotopically resolved mass fluxes will help refine models for drying mechanisms in porous media.

Heritage science is a contemporary interdisciplinary subject that seeks to understand change and risk to preserve and maximize the cultural and socio-economic benefits of heritage for future generations.^1^ Heritage materials are often very difficult to study, requiring state-of-the-art physical and chemical analysis methods, and the field has hugely benefited from the development and application of new analytical techniques.^2−4^ One significant research area concerns the built historic environment and its conservation, and in particular how to mitigate the effects of moisture retention and transport within both traditional and new building materials. With this aim in mind, here we show how cavity ring-down spectroscopy (CRDS) can be used to monitor both the moisture content and dynamics in small samples of limestone (ca. ≤5 g), a sedimentary rock mainly composed of calcium carbonate, CaCO_3_, which is a commonly used building stone across the globe.^5−9^ This widespread use is in part due to the relative ease that it can be worked and shaped, as well as a result of its perceived durability.^10^

The longevity of historic buildings constructed from limestone indicates that deterioration is a slow and gradual process, mainly occurring via the karst (or karstification) process,^11^ which is the dissolution of limestone by rainwater, normally acidified by the uptake of CO_2_ or organic acids. However, in polluted metropolitan environments, dissolution markedly increases through wet and dry deposition processes. Water is a key agent in most physical, chemical, and biological decay processes;^12^ there are various ways water can enter buildings, through rising damp from the soil or through the pore network, resulting in the mechanical damage of the stone material due to transformational stress and various physical cycles, for example, freeze–thaw.^13^ In addition, water acts as a transport medium and carries contaminants into the stone,^14^ leading to chemical attack, salt crystallization, and biodeterioration, resulting in further mechanical damage.^15−17^ Signatures of deterioration include flaking, powdering, and other forms of material loss, but the majority of these can be mitigated through the use of consolidants. Clearly, a consolidation treatment should not only improve the mechanical strength of the sample but also be compatible with the treated substrate and resistant to different damage mechanisms.^18^ For the ongoing development and optimization of both organic and inorganic chemical treatments, it is imperative to know the concentration and location of water within the stone as well as the net water flux to understand these coupled physicochemical decay processes.^19−21^

This article reports the first use of CRDS as a high-resolution, nondestructive quantitative laboratory based method to monitor moisture content and fluxes for samples of limestone. In particular, the sensitive and selective nature of CRDS when combined with isotopic enrichment of water samples allows determination of the drying kinetics within environments of varying relative humidity (% RH), key quantitative information in the area of heritage conservation. CRDS references each measurement of the ring-down time to that for an empty cavity, and as such allows absolute densities to be readily and directly determined with no instrument calibration required, as demonstrated in the equivalence of the spectroscopically and gravimetrically determined masses. In this article, the principles of the near-IR CRDS instrument that has been developed are outlined, before presentation of the proof-of-principle data from which the time-dependent water contents of limestone samples are determined. Two stages of drying are clearly observed, in agreement with conventional theories for the drying of porous materials.^22^ The reproducibility and accuracy of the measurements are also discussed, before the simultaneous monitoring of water and its isotopologue, HDO, is demonstrated in the evaluation of moisture fluxes under conditions of varying % RH. Prospects for this method are briefly discussed in the Conclusion.

## Materials and Methods

### Cavity Ring-Down Spectroscopy

#### Theory

Absorption spectroscopy allows the identification of species based on their unique set of quantum-mechanical energy levels. When light passes through an absorbing species, the intensity of light decreases as a result of the resonant absorption of energy, and this attenuation in intensity is quantified by the Beer–Lambert law, *I*_tr_(ν) = *I*_0_(ν) exp[−α(ν)*d*], where *I*_0_(ν) and *I*_tr_(ν) are the incident and transmitted light intensities at frequency ν, respectively, α(ν) is the frequency-dependent absorption coefficient of the species, and *d* is the path length. When the absorption is by a single species, the absorption coefficient is related to the number density, *N*, by α(ν) = *N*σ(ν), where σ(ν) is the (species-specific) absorption cross section. Thus, absorption measurements yield absolute number densities.

The absorption intensity, *I*_abs_(ν), is given by the difference , and it is clear that for a given absorption intensity, *I*_abs_(ν), increasing the path length, *d*, over which the light interacts with the sample allows smaller number densities to be measured, increasing the sensitivity of the absorption measurement. CRDS increases the effective path length over which the light can interact with the sample by enclosing the sample between two (or more) highly reflective mirrors.^23,24^ This work utilizes continuous-wave (cw) CRDS in which cw laser radiation is focused into an optical cavity formed from two dielectric mirrors, aligned such that cavity modes are readily and regularly excited.^25−28^ The light intensity exiting the cavity is monitored, and when a certain threshold is reached, the laser radiation is switched off, causing the light intensity within the cavity to decay exponentially. This decay is characterized by a ring-down time, τ_0_, which is the time taken for the light intensity to decrease to 1/*e* of its original value, *I*_0_. If the only losses are due to the imperfect reflectively of the mirrors, then this decay time is given by , where *c* is the speed of light and *R* the reflectivity of the mirrors. In the presence of an absorbing species, such as water (and in the absence of other loss processes), the rate of decay is faster (i.e., the ring-down time of the cavity is reduced) because light is lost from the cavity, both by transmission through the mirrors and by absorption by the molecular species under investigation, resulting in a modified ring-down time, τ. The difference in the reciprocals of the ring-down times allows the determination of the absorption coefficient, α(ν):
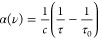
1and hence the number density of the absorbing species.

#### Instrument Design

**Figure 1:**
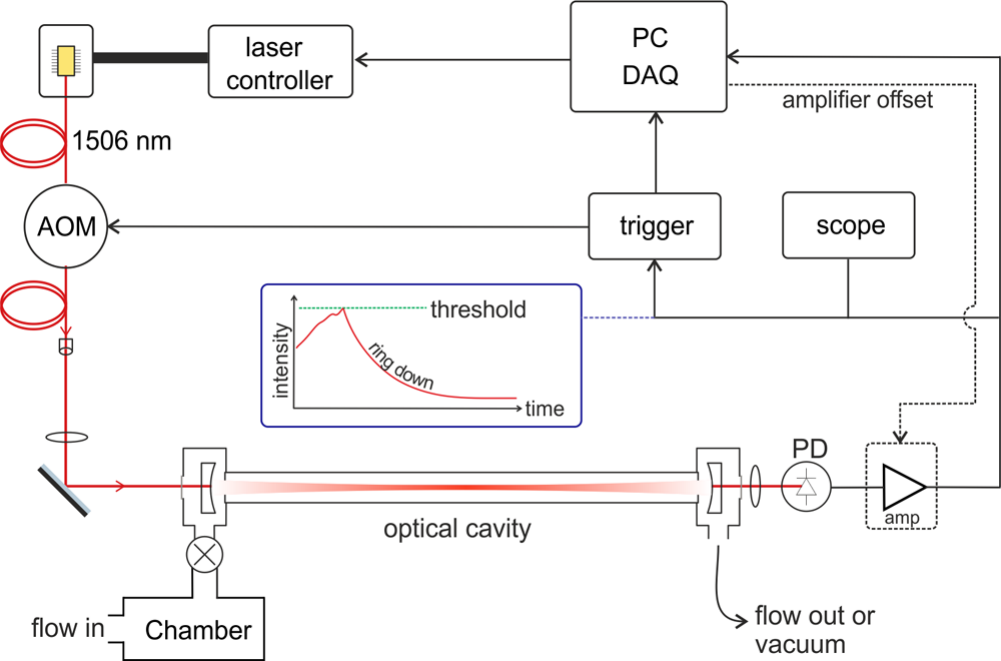
A schematic diagram of the CRDS used to measure H_2_O released from limestone. AOM = acousto-optic modulator; PD = photodetector; amp = amplifier; DAQ = data acquisition system.

The near-IR CRDS instrument used in this work is depicted in Figure 1. The laser used is a cw fiber-coupled distributed feedback diode laser (NTT Electronics NLK1S5GAAA, λ = 1506 nm), which is temperature- and current-controlled (Thorlabs TED200C and LDC205C). The near-IR region was selected as it is a technologically highly developed wavelength region with relatively strong H_2_O and HDO absorptions. The laser beam passed through a fiber-coupled acousto-optic modulator (AOM) (Gooch & Housego Fiber-Q A35) and then through an adjustable fiber collimation package (Thorlabs CFC-8X-C). Two mode-matching lenses, *f* = 100 mm and *f* = 50 mm (Thorlabs LB1676-C and LB1471-C, respectively), were used to focus the beam into the cavity. To form the cavity, two highly reflective mirrors (Layertec, *R* ≈ 0.9999, radius of curvature = 1 m) were held in kinematic mounts (Thorlabs Polaris-K1-2AH) housed in stainless steel enclosures, a distance of 86 cm apart. One face of each enclosure is a transparent window (Thorlabs WG41050-C), whereas the other face is connected to a borosilicate glass tube with an outer diameter of 18 mm. The typical limit of detection for this apparatus is α_min_ ≲ 1 × 10^–9^ cm^–1^ in <10 s, where α_min_ is the minimum detectable absorption coefficient. In the precise spectral region we use, around 6638 cm^–1^, this corresponds to approximately 500 ppb of water at atmospheric pressure.

The light transmitted through the cavity is focused onto a photodetector (Thorlabs DET410 InGaAs photodiode), and the signal is amplified (current amplifier Femto DLPCA-200) and acquired using a data acquisition system (National Instruments NI-USB 6356). When the transmitted signal reaches a predetermined threshold intensity, a signal is sent to a custom-built trigger box, which both switches off the AOM (and thus stops light entering the cavity) and triggers the acquisition system. A LabVIEW (National Instruments) program has been designed to control the experiment and fit the exponential decays (of light intensity) to determine values for τ. The same program controls an offset voltage on the amplifier that depends on the previous measured ring-down time (RDT) to ensure that the rate of acquisition remains relatively constant (∼10 Hz), and that only the low-order modes are triggered by the acquisition. The program has the capability to acquire absorption spectra using temperature tuning of the cw diode laser, as well as a dual-wavelength switching function.^29^ (The temperature dependence of the wavelength under operating conditions is shown in the Supporting Information.) This function automatically switches the laser between two set temperatures, providing high-resolution monitoring of two transitions of choice. To ensure the occurrence of regular CRD events, the laser is modulated at a frequency of 5 Hz and amplitude 200 MHz (an amplitude a little higher than the free spectral range of the cavity, but not too large compared to the pressure-broadened spectral line widths).

Inlets through the stainless steel enclosures allow control and monitoring of the environment inside the cavity, with pressure monitored using a pressure gauge (Leybold Vacuum CTR90, measurement range = 0.1–1000 Torr). Experiments are carried out in a N_2_ (BOC Oxygen Free Nitrogen, 99.998%) gas flow with preselected levels of % RH, controlled with calibrated mass flow controllers (MKS 1479A Mass-Flo and MKS PR 4000B-S) and rotameters. The baseline RDT in a dry N_2_ atmosphere is τ_0_ ≈ 150 μs (corresponding to a cavity finesse of ∼160000). A separate stainless steel sample chamber (13 × 8 × 7 cm) is attached to one of the enclosures, separated by a valve. This allows a background spectrum of the residual water vapor in the cavity to be taken, providing a measure of τ_0_, and allows the sample conditions to be treated independently of the optical cavity.

### Stone Samples: Clipsham Limestone

Clipsham limestone is exclusively used in this work as an exemplar. Clipsham limestone is a British limestone from the Lincolnshire Limestone formation (Middle Jurassic, Bajocian), is composed of cross-bedded oolites (characteristic spherical grains) with occasional herringbone cross-bedding,^30,31^ and is a very pale cream and buff color. It is a coarse-grained ooidal and shell fragmental grainstone and was deposited in a shallow marine carbonate shelf environment.^32^ Clipsham limestone has been widely used in a variety of buildings, both for construction^33^ and repair.^34,35^ Mercury intrusion porosimetry^36^ was carried out to characterize the internal structure of the stone. The results of this analysis are available in Figure S1 of the Supporting Information. The samples have an open porosity of 20.39% and density of 2.69 g cm^–3^. The pore size distribution is unimodal with a median pore diameter of 1.18 μm. A sample size of ca. 25 × 25 × 5 mm was selected to allow relatively fast drying experiments, and a total of four different Clipsham limestone samples were used in this study.

### Experimental Procedure

For accurate analysis, it was essential to determine the water continuum contribution. To achieve this, a series of CRD spectra of increasing % RH were acquired over the range 6636.34–6640.11 cm^–1^ at a constant temperature of 294 K and a pressure of 767 Torr (ca. atmospheric pressure). The % RH was changed by passing increasing amounts of a calibrated flow of N_2_ through a water bubbler and mixing with a dry flow of N_2_ to ensure a total flow of 1.5 standard liters per minute (slm) remained.

To monitor the release of water from the limestone samples, the samples were first immersed in distilled water for a minimum of 2 days. This is to ensure the samples are saturated, and this time was determined as the minimum required time following a pilot study into the influence of soaking times on drying kinetics. Upon removal, excess water was sponged off the sample, and its wet mass, *m*_w_, recorded. The sample was then placed in the sample chamber, the valve opened to the main chamber, and a constant flow of 1.5 slm N_2_ passed over the sample into the cavity. The laser was tuned to the center of the H_2_O 10,3,7 (021) ← 11,3,8 (000) transition at 6638.91 cm^–1^ (spectroscopic nomenclature is defined in the Supporting Information) and the average RDT continuously measured. The procedure was stopped when the measured RDT returns to τ_0_. The dry mass of the sample, *m*_d_, was also recorded.

## Results and Discussion

### Drying Kinetics under Dry Flows

**Figure 2:**
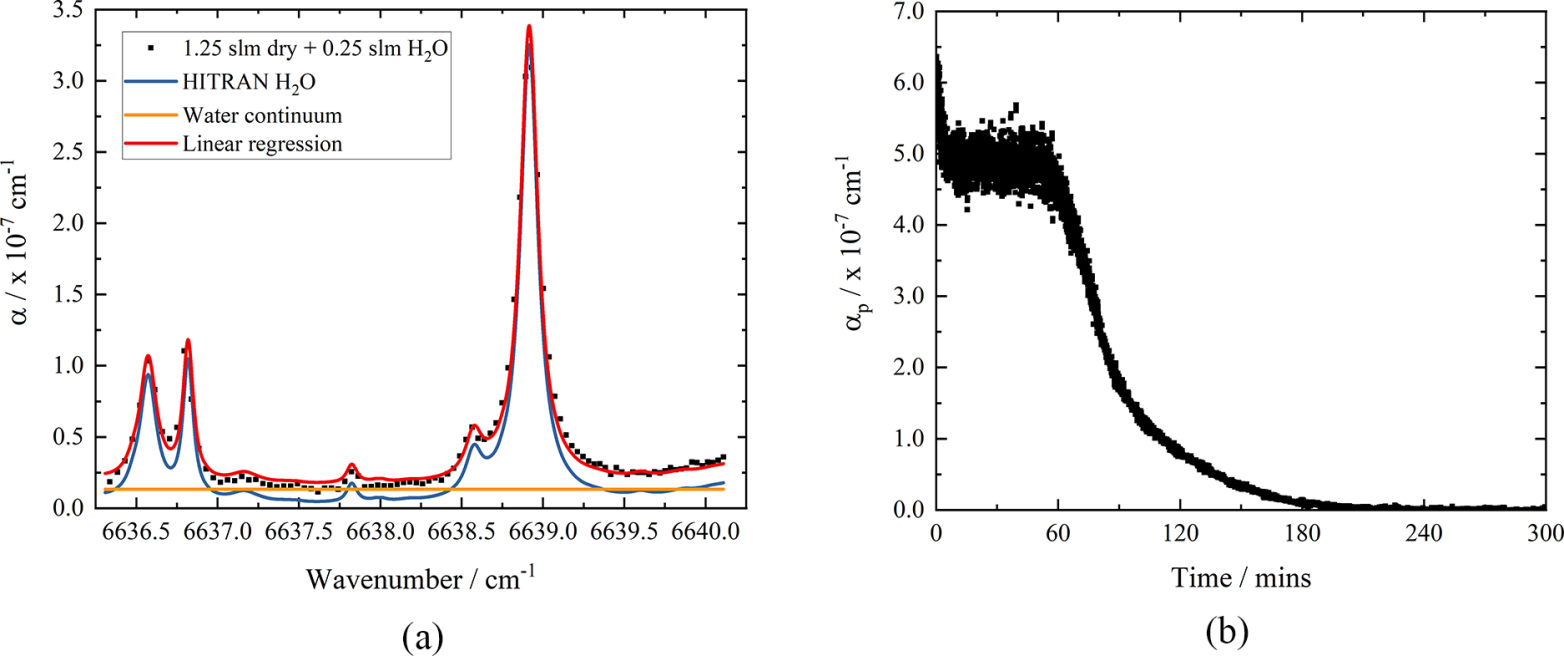
(a) Absorption spectrum of 0.25 slm wet N_2_ in 1.25 slm dry N_2_ (% RH = 7.5) over the range 6636.34–6640.11 cm^–1^ at *T* = 294 K and *P* = 767 Torr. A linear regression (*R*^2^ = 0.98) of the HITRAN simulation of the absorption coefficient for H_2_O (blue) over the same range and water continuum (orange) is shown. (b) A plot showing α_p_ as a function of time for a 1.5 slm N_2_ flow over the Clipsham limestone ca. 25 × 25 × 5 mm sample. α_p_ is obtained by tuning the laser to the center of the H_2_O 10,3,7 (021) ← 11,3,8 (000) transition at 6638.91 cm^–1^ and continuously measuring the RDT.

Using the CRD spectra obtained for increasing % RH, for each wavelength, α is calculated (eq 1) from the average RDT of 10 ring-down events. Figure 2a shows the resultant CRD absorption spectrum of 0.25 slm wet N_2_ mixed with 1.25 slm dry N_2_ (equivalent to 7.5% RH). The spectrum consists of well-resolved absorption features superimposed upon an underlying continuum absorption,^37^ both of which depend upon the concentration of water molecules present. Using a linear regression, the absorption spectrum was simulated with HITRAN data^38^ of the absorption cross section of water at atmospheric pressure and a contribution representing the water continuum, termed α_offset_. Using the increasing % RH spectra, the relationship between the water continuum contribution and number density of water was determined, allowing this to be accounted for in the following investigation. For example, for a 0.25 slm wet flow, as shown in Figure 2a, α_offset_ = 8.8 × 10^–9^ ± 1.2 × 10^–9^ cm^–1^.

The time-dependent release of water from limestone samples was monitored using the method described above. Using eq 1, the absorption coefficient at the line center, which we denote as α_p_, is determined as a function of time as shown in Figure 2b. Experiments in which extended absorption spectra were recorded as the stone dries under the same conditions described above were also performed to confirm the results obtained with monitoring at one or more fixed wavelengths, *vide infra*.

Using the spectra from a range of % RH values, the pressure-broadening parameter for the H_2_O transition was evaluated and used to determine the peak absorption cross section, σ_p_, at the line center from the integrated cross section listed in HITRAN,^38^ assuming a Voigt line shape (this improves on the fit to the spectra in Figure 2a, increasing the goodness-of-fit parameter *R*^2^ from 0.98 to 0.998). Once σ_p_ is known, the number density of water molecules is calculated using α_p_ = *N*σ_p_. Here, a small correction required to take into account the predetermined water continuum absorption is applied. To calculate the mass of water released, and allow determination of the drying kinetics, the following procedure is used.

If *n*_t_ is the total number of water molecules present in the system at time *t*, *V* is the volume of the system, ϕ_in_(*t*) and ϕ_out_(*t*) are the molecular (water) flux in and out of the cavity, then for a given volumetric flow rate (*f*) the following relationship can be constructed:
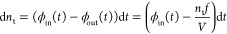
2We measure the number density, *N*, which is equal to *n*_t_/*V*, the time-dependent concentration, and want to determine *∫ϕ*_in_ d*t*, from which the total mass of water released from the stone can be calculated. From eq 2, the cumulative mass of water released by the limestone sample to a time *t* is then given by
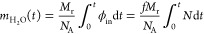
3where *N*_A_ is Avogadro’s number and *M*_r_(H_2_O) = 0.018 kg mol^–1^. For the data shown in Figure 2b, the spectroscopically determined total mass released, , is 0.361 ± 0.003 g. The total mass change determined by gravimetric analysis (Salter Brecknell ESA-300, max. 300 g, δ = 0.005 g) is 0.360 ± 0.005 g, showing good agreement with the spectroscopically determined value.

**Figure 3:**
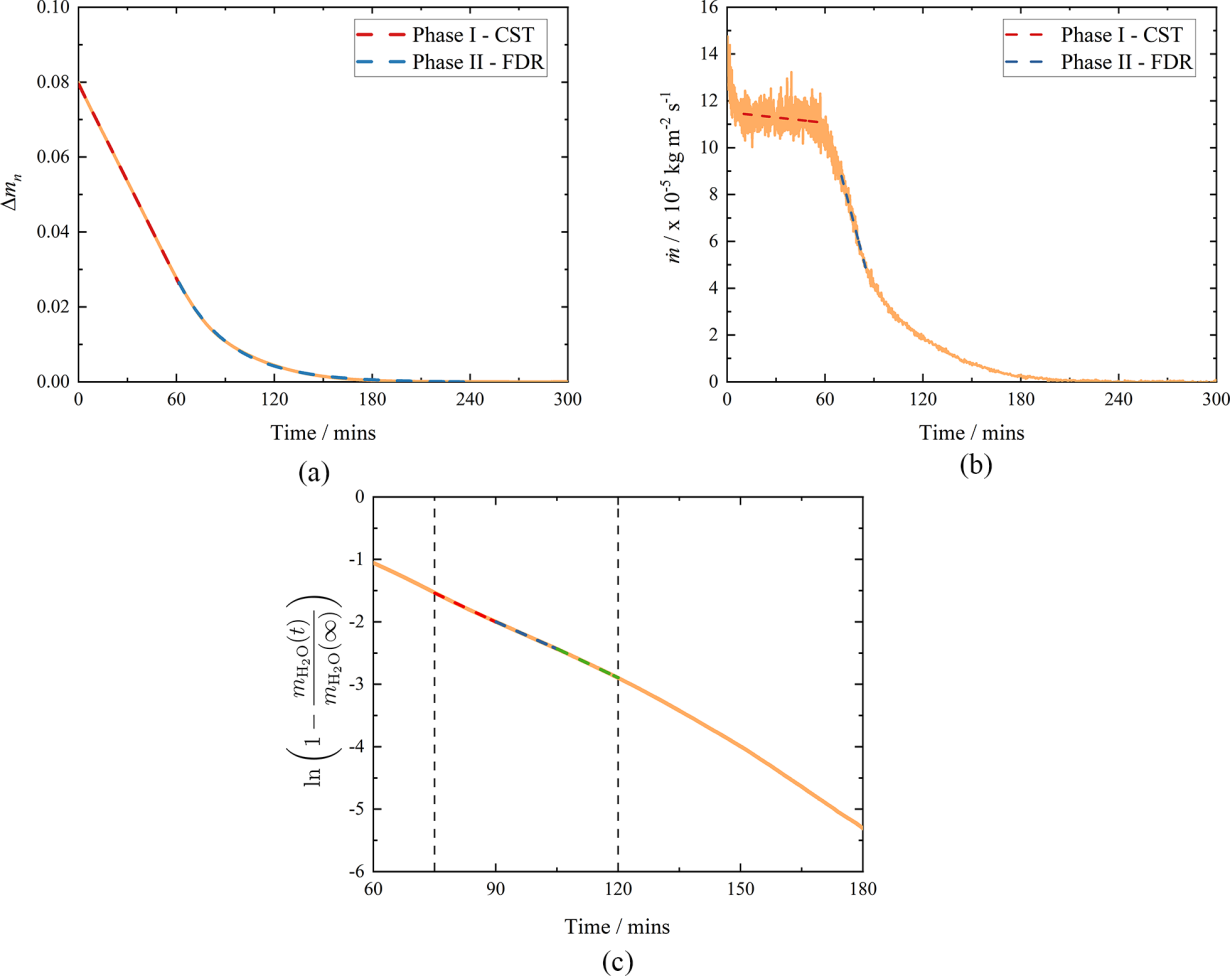
(a) Normalized mass difference as a function of time for a Clipsham limestone ca. 25 × 25 × 5 mm sample drying under a constant dry 1.5 slm N_2_ flow. Phase I has been fitted with a linear function, whereas phase II has been fitted with an exponential function. (b) A plot of the water flux, *ṁ*, as a function of time, showing a linear fit to phase I, allowing *ṁ*_CST_ to be determined. (c) A plot of  against time, allowing determination of *D*_II_. The region of linear fit is indicated by two vertical lines.

The normalized mass difference, Δ*m*_n_(*t*), is defined as
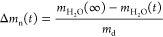
4Figure 3a shows Δ*m*_n_(*t*), illustrating the expected two-phase drying process of porous materials.^12,39^ Phase I is defined as the constant drying rate period (CST) during which the rate of evaporation is controlled by the external conditions and does not depend on the transport processes within the material.^40^ Water is supplied to the evaporation pores at the surface by flow through connected pathways, resulting in the air at the surface being fully saturated; diffusion from the material to the air occurs down a constant concentration gradient that controls the rate of evaporation.^41^ As the drying proceeds further, flow occurs through increasingly disconnected pathways, and the surface and mean water contents decrease such that the air at the surface is not saturated. Eventually, the unsaturated capillary flow within the sample can no longer occur at a rate sufficient to maintain a constant evaporation rate.

Data in phase I are commonly fitted with a linear function, as shown in Figure 3a. The mass flux, , is defined as
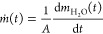
5where *A* is the total surface area of the sample and  is the gradient of a linear fit to the data. Thus, for Figure 3b, the constant mass flux for phase I,  kg m^–2^ s^–1^. The critical time at which phase I ends, *t*_c_, was determined to be the intercept between two tangents fitted to *ṁ*(*t*), shown as dashed lines in Figure 3b. On the basis of the analysis described by Castro and Coelho Pinheiro,^42^ an iterative approach was used to determine *t*_c_: the intercept of the two tangents was determined, and this value was then set as the upper limit for the linear fit to phase I and the process repeated until a constant *t*_c_ was determined.

As noted above, phase II drying begins as the rate of evaporation starts to fall, known as the falling drying rate period (FDR), and is regulated by the unsaturated flow within the porous material. In Figure 3a, this phase has been fitted with an exponential function. Drying in this phase can be described by Fick’s second law, (*∂C*/*∂t*) = *D*_II_(∂^2^*C*/*∂x*^2^), which relates the change in the moisture concentration, *C*, with time to the spatial variation of its concentration gradient; here, *x* is the distance over which moisture transfer occurs, as measured from the bottom face of the sample, and *D*_II_ is the phase II diffusivity. Assuming the moisture is uniformly distributed through the porous sample, and integrating over the sample thickness *L*, allows *m*(*t*) to be written as^43^
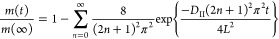
6In this study, we found that stage II drying begins when the moisture content falls below a critical saturation value of ∼30%.^21^ In these later stages of drying, only the first term in eq 6 needs to be considered,^44^ and *D*_II_ can therefore be determined from a linear relationship between the logarithmic terms and time:
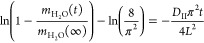
7

At even longer times (>135 min) slight deviations to this are seen, but we note that by this time 97% of the water has been released.

This analysis of our data is illustrated in Figure 3c: the average gradient is equal to −5.1 × 10^–4^ ± 2 × 10^–5^ s^–1^, and using *L* = 3.8 × 10^–3^ m yields *D*_II_ = 3.0 × 10^–9^ ± 1 × 10^–10^ m^2^ s^–1^. Using this procedure on plain cement paste, Bakhshi and Mobasher^45^ obtained a diffusivity of 3.33 × 10^–9^ m^2^ s^–1^, whereas Zaknoune et al.^46^ reported *D*_II_ values 2–3.5 × 10^–9^ m^2^ s^–1^ for lime binder and 4.5–6.7 × 10^–9^ m^2^ s^–1^ for cellular concrete. Thus, the values obtained in this work seem to be in good agreement with those reported in the literature for similar materials.

To test the reproducibility of the measurement, and also the variation between samples of the same limestone, this experiment was repeated with four ca. 25 × 25 × 5 mm Clipsham limestone samples. Table 1 summarizes the drying kinetics for the four samples. As expected, there are slight variations due to differences between the samples but overall good agreement between the different kinetic parameters determined. The drying kinetics are also depicted in Figure S2 in the Supporting Information.

**Table 1:** Comparison of Drying Kinetics Using Different Samples of Clipsham Limestone, All ca. 25 × 25 × 5 mm^a^

sample	C1	C2	C3	C4
*m*_H_2_O_(∞) (g)	0.361 ± 0.003	0.400 ± 0.003	0.393 ± 0.003	0.408 ± 0.003
gravimetric mass (g)	0.360 ± 0.005	0.390 ± 0.005	0.390 ± 0.005	0.410 ± 0.005
*t*_c_ (min)	61.50	67.18	66.11	72.89
critical saturation value (%)	33	35	31	32
*ṁ*_CST_ (kg m^–2^ s^–1^)	1.13 × 10^–4^ ± 9 × 10^–6^	1.09 × 10^–4^ ± 9 × 10^–6^	1.16 × 10^–4^ ± 9 × 10^–6^	1.08 × 10^–4^ ± 9 × 10^–6^
*D*_II_ (m^2^ s^–1^)	3.0 × 10^–9^ ± 1 × 10^–10^	2.8 × 10^–9^ ± 2 × 10^–10^	3.2 × 10^–9^ ± 4 × 10^–10^	3.0 × 10^–9^ ± 2 × 10^–10^

a Note:  = spectroscopic mass; *t*_c_ = critical time; *ṁ*_CST_ = constant mass flux for phase I; *D*_II_ = phase II diffusivity.

### Isotope Detection for Moisture Flux Determination

This work utilizes a cw diode laser, an easily tunable and controllable device with high spectral purity, which is readily able to distinguish between different isotopologues that themselves exhibit unique absorption spectra. Therefore, preparing a sample in isotopically enriched water and subsequently detecting the presence of HDO or D_2_O allows the release of water from within the limestone to be decoupled from the adsorption and desorption of ambient water in the environment. For example, the natural abundance of HDO is very low (ca. 0.031%), and therefore it can be assumed that any HDO measured has been released from the sample. The absorption cross sections of D_2_O and HDO transitions in the spectral region of interest are approximately an order of magnitude larger than those for H_2_O,^38^ and thus a 10:1 mixture of H_2_O and D_2_O (Sigma-Aldrich, 99.9% atom D) was used for immersion; these species undergo isotopic substitution to produce an equilibrium mixture containing HDO, H_2_O, and D_2_O,^47^ in approximately the following ratio 15:84:1.

**Figure 4:**
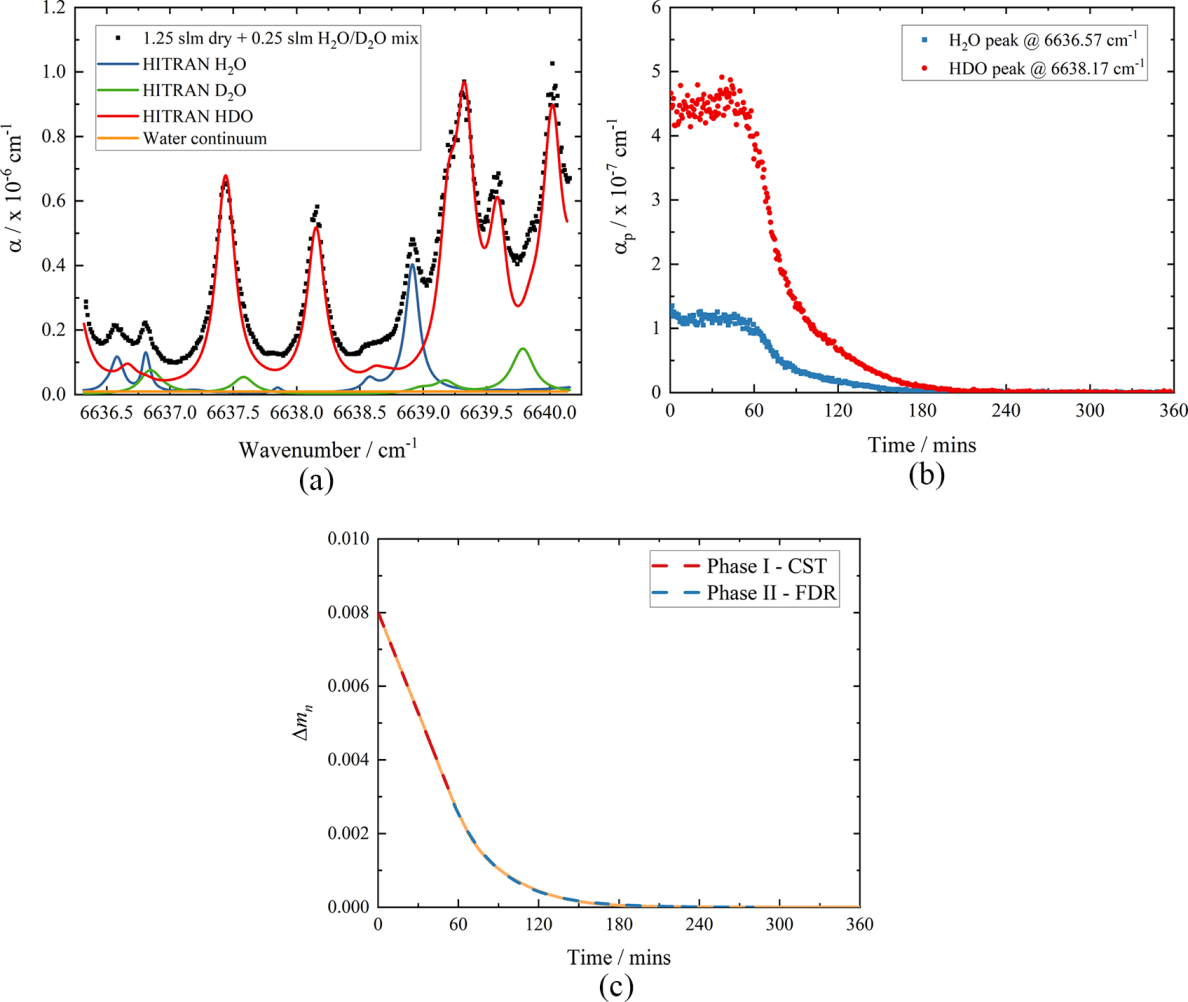
(a) Absorption spectrum of 1.25 slm dry N_2_ mixed with 0.25 slm wet N_2_ (% RH = 7.5) flowed through the 10:1 mixture of H_2_O and D_2_O over the range 6636.34–6640.11 cm^–1^ at *T* = 295 K and *P* = 770 Torr. A linear regression of the HITRAN simulation of the absorption coefficient for H_2_O (blue), D_2_O (green), and HDO (red) over the same range and water continuum (orange) is shown (contributing to a global fit, *R*^2^ = 0.99).(b) A plot showing α_p_ as a function of time for a 1.5 slm N_2_ flow over the Clipsham limestone ca. 25 × 25 × 5 mm sample that had been immersed in a 10:1 (H_2_O/D_2_O) mixture. (c) HDO normalized mass difference as a function of time for Clipsham limestone ca. 25 × 25 × 5 mm sample drying under a constant 1.5 slm N_2_ flow.

Figure 4a shows a CRD spectrum obtained with a 1.25 slm dry N_2_ flow mixed with a 0.25 slm wet N_2_ flow (% RH = 7.5) passed through the 10:1 mixture, taken over the region 6636.34–6640.11 cm^–1^ at a constant temperature of 295 K and at a pressure of 770 Torr. Using the same linear regression program as before, the spectrum was fitted with HITRAN simulations of the three dominant isotopologues along with the water continuum, as shown in Figure 4a. From this, it is clear that, first, the absorption at 6638.17 cm^–1^ is due purely to the HDO 8,6,2(210) ← 9,6,3(000) transition; second, the contribution from D_2_O is negligible and therefore will not need to be taken into account when monitoring the water released from the limestone samples; and third, the measured absorptions at the H_2_O transition frequencies have contributions from HDO, and therefore this must be taken into account when determining the mass of H_2_O released.

To monitor the release of both HDO and H_2_O from limestone to confirm that HDO can be used to accurately measure limestone drying kinetics, a wavelength switching routine was used. The LabVIEW control program was set to switch between the center of the H_2_O 6,0,6(031) ← 7,0,7(010) transition at 6636.57 cm^–1^ and the center of the HDO 8,6,2(210) ← 9,6,3(000) transition at 6638.17 cm^–1^, with a 10 s pause between acquisitions. The same experimental procedure as described for H_2_O was used, except the samples were immersed in the 10:1 mixture of H_2_O and D_2_O for 2 days (instead of distilled water). Figure 4b shows a plot of α_p_ for both H_2_O and HDO for a Clipsham limestone ca. 25 × 25 × 5 mm sample dried under a 1.5 slm dry N_2_ flow. From the linear regression shown in Figure 4a, it was determined that the HDO contribution to the absorption at 6636.57 cm^–1^ is 16.5% of that measured at 6638.17 cm^–1^, and this has been taken into account to produce the α_p_ values for H_2_O shown in Figure 4b. We note that the relative abundances of the isotopologues emanating from the stone can be subtly, and reproducibly, different from that in the isotopically enriched water. This effect has its origin in the residual water content of the limestone samples and occurs in samples that had not previously been subjected to an isotopic mixture—the stone samples are not oven-dried in these proof-of-principle experiments, and as such there are small amounts of residual water content in the nominally dry stone that can cause small shifts in the chemical equilibrium between isotopologues.

Using eq 4, Δ*m*_n_ for HDO released was calculated, and Figure 4c shows a plot of Δ*m*_n_(*t*). As expected, this shows the two-phase drying behavior as previously seen in Figure 3a; however, we note that Δ*m*_n_ is an order of magnitude smaller in this case because of the smaller amount of HDO initially present. The data were analyzed using the same procedure described for the H_2_O drying kinetics and produced the parameters listed in Table 2. A comparison of these values to those previously presented for a stone immersed in pure H_2_O shows good consistency, with a lower amount of H_2_O measured due to a lower initial amount of pure H_2_O present in the stone. Because of the differences in the total masses of H_2_O and HDO released from the sample, and to allow direct comparison of the constant flux, *m*(*t*) can be normalized to have a maximum of 1. From this, the normalized mass flux, *ṁ*_N_, can be calculated and the normalized constant mass flux for phase I, *ṁ*_NCST_, can be determined. For H_2_O, *ṁ*_NCST_ is 0.332 ± 3 × 10^–4^ m^–2^ s^–1^ and for HDO it is 0.334 ± 3 × 10^–4^ m^–2^ s^–1^. This good agreement between these values gives confidence that HDO can be used to accurately monitor limestone drying kinetics. The consistency between *D*_II_ for H_2_O and HDO, as shown in Table 2, also supports the use of HDO to monitor limestone drying kinetics. We further note that the observed difference in *t*_c_ for H_2_O and HDO is due to the cycling of the switching between transitions, that is, the difference in *t*_c_ values is within one period of the diode laser switching cycle, and not due to any physical difference in kinetic behavior of each isotopologue.

**Table 2:** Comparison of Drying Kinetics for H_2_O and HDO Released from a Clipsham Limestone ca. 25 × 25 × 5 mm Sample under a 1.5 slm Dry N_2_ Flow^a^

species	H_2_O	HDO
*m*(*∞*) (g)	0.305 ± 0.004	0.0357 ± 0.0003
*t*_c_ (min)	56.75	57.11
*ṁ*_CST_ (kg m^–2^ s^–1^)	1.01 × 10^–4^ ± 9 × 10^–6^	1.19 × 10^–5^ ± 9 × 10^–6^
*D*_II_ (m^2^ s^–1^)	2.9 × 10^–9^ ± 3 × 10^–10^	3.2 × 10^–9^ ± 4 × 10^–10^

a Note: *m*(*∞*) = spectroscopic mass; *t*_c_ = critical time; *ṁ*_CST_ = constant mass flux for phase I; *D*_II_ = phase II diffusivity.

**Figure 5:**
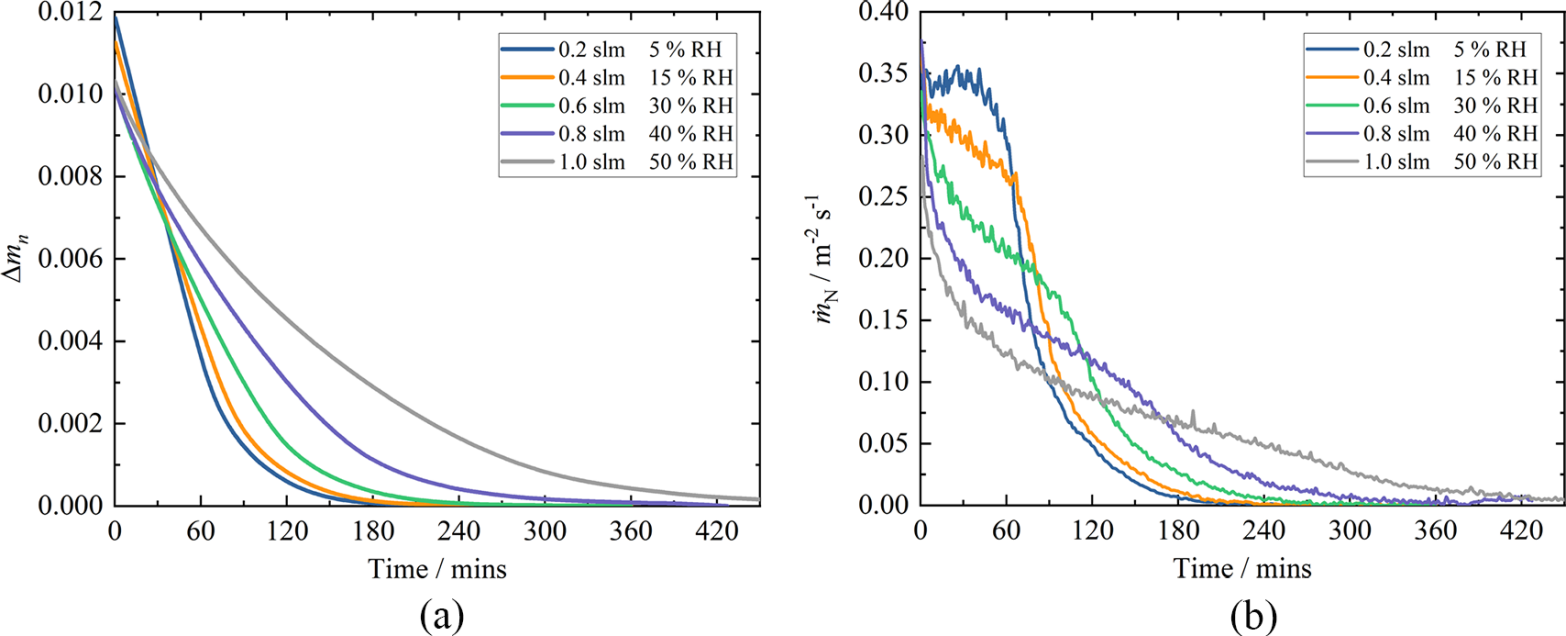
(a) A plot of the HDO normalized mass difference as a function of time with increasing % RH for the same ca. 25 × 25 × 5 mm Clipsham limestone sample. The total combined flow was 1.5 slm N_2_. (b) A plot of the HDO normalized mass flux, *ṁ*_N_, as a function of time with increasing % RH for the same ca. 25 × 25 × 5 mm Clipsham limestone sample.

Clearly, HDO can be used to accurately monitor limestone drying kinetics, but the main advantage it offers is the ability to determine drying kinetics under different environmental conditions. Specifically, this technique allows limestone drying to be monitored under conditions of increasing % RH. Following the same procedure as described before, experiments were conducted in which the composition of the “drying” gas was changed in steps from 0.0 to 1.0 slm wet N_2_ and then combined with dry N_2_ to make a constant total gas flow of 1.5 slm. Absorption spectra were taken at each composition and were analyzed to determine the number density of water, from which, using the ideal gas law and saturated vapor pressure of water,^48^ the % RH can be calculated (this is also done without the sample present to characterize the wet flow). Thus, changing from 0.0 to 1.0 slm wet N_2_ corresponds to increasing the humidity from 0 to 50% RH. All other aspects of the experiment were kept the same, and the results are shown in Figure 5.

Figure 5a shows Δ*m*_n_(*t*) for the HDO released, and as previously seen in Figure 4c, it is an order of magnitude smaller than that for H_2_O because of the smaller amount of HDO initially present. However, in Figure 5a, it is apparent that as the % RH increases, the two-phase drying behavior becomes less distinct in the HDO drying curves. Initially, at the lower wet flows ranging from 0.0 to 0.6 slm, Δ*m*_n_(*t*) does show this two-phase process, although with a decrease in the gradient of phase I and a concomitant increase in *t*_c_ as % RH increases. However, for the higher wet flows of 0.8 and 1.0 slm, there are no longer two distinct phases. This change in behavior is more apparent in the plot of normalized mass flux, *ṁ*_N_, which is shown in Figure 5b and the parameters reported in Table 3.

**Table 3:** Comparison of HDO Drying Kinetics with Increasing Humidity for a Clipsham Limestone ca. 25 × 25 × 5 mm Sample^a^

wet flow (slm)	0.0	0.2	0.4	0.6	0.8	1.0
% RH	0	5	15	30	40	50
*t*_c_ (min)	57.11	55.85	68.66	100.44	145.55	333.76
*ṁ*_NCST_ (m^–2^ s^–1^)	0.334 ± 4 × 10^–4^	0.341 ± 4 × 10^–4^	0.288 ± 7 × 10^–4^	0.212 ± 1 × 10^–3^	0.138 ± 8 × 10^–4^	0.058 ± 5 × 10^–4^
*D*_II_ (m^2^ s^–1^)	3.2 × 10^–9^ ± 4 × 10^–10^	3.2 × 10^–9^ ± 4 × 10^–10^	2.9 × 10^–9^ ± 3 × 10^–10^	2.5 × 10^–9^ ± 2 × 10^–10^	1.6 × 10^–9^ ± 9 × 10^–11^	1.1 × 10^–9^ ± 4 × 10^–12^

a Note: % RH = % relative humidity; *t*_c_ = critical time; *ṁ*_NCST_ = normalized constant mass flux for phase I; *D*_II_ = phase II diffusivity.

As the % RH increases, there is a resulting decrease in *ṁ*_NCST_, as predicted by Fick’s first law,^40^ and this decrease in evaporation rate means that the time taken before disconnected pathways form is longer, resulting in an increase in *t*_c_. The critical time, *t*_c_, was determined using the same procedure as described before for pure H_2_O; however, in Figure 5b, it can be seen that the clear change from phase I to phase II as seen for 0.0 and 0.2 slm becomes less defined with increasing humidity, and therefore this leads to questions over defining discrete drying phases and *t*_c_. Furthermore, with increasing humidity, an initial decrease in *ṁ*_N_ becomes apparent within the phase I drying period, and the time scale over which this occurs lengthens from approximately 10 min for 0.0 slm to 30 min for 1.0 slm wet N_2_. Franzen and Mirwald^12^ proposed that the origin of this initial sharp decrease in *ṁ*_N_ is due to vaporization, which is not influenced by the stone surface. This vaporization, therefore, may be from a water layer on the surface of the stone, and with increasing humidity, this water layer increases in thickness, resulting in a longer time scale and a greater observed decrease in *ṁ*_N_.

From both the data presented in Table 3 and Figure S3 of the Supporting Information, it can be seen that *D*_II_ also decreases with increasing % RH. As previously stated, phase II is determined by unsaturated flow within the material and should be independent of the external environment. However, these results imply that the external conditions influence the late stage drying behavior. We note that the value of *D*_II_ at the highest % RH is broadly consistent with the self-diffusion coefficient of liquid water, reflecting the importance of water at the solid–gas interface, even at these later stages. This observation and the interpretation of late stage drying requires further investigation and quantitative modeling.

## Conclusion

This article presents the first use of cavity ring-down spectroscopy with dual-wavelength switching to quantitatively monitor the drying kinetics of Clipsham limestone. The spectroscopically deduced masses are in good agreement with those measured gravimetrically, and the drying kinetics are well described by the classic two-phase model when dry flows are employed. The ability to switch quickly and reproducibly between multiple wavelengths allows different isotopologues to be monitored, and there is excellent agreement between the measured drying kinetics and diffusivity parameters when monitoring H_2_O and HDO from samples that have been isotopically enriched. When the relative humidity of the drying gas was increased, there was an observed decrease in the phase I normalized mass flux and an increase in its time scale, as predicted using Fick’s law. A clear decrease in phase II diffusivity with increasing relative humidity was also observed.

In the future, it will be interesting to use the CRDS technique to investigate the evaporation of water isotopologues under varying wet depositions within atmospheric simulation chambers of the type presented by Chabas et al.^49^ Further work will also investigate the influence of temperature on the drying kinetics of different classes of limestone and other porous materials that have been subject to treatment with consolidants. Finally, we note that the CRDS technique can be used to readily couple the detection of water and its isotopologues with the sensitive and selective detection of other trace gas species and pollutants; such measurements will further enhance our understanding of decay mechanisms within these heritage materials and strategies for ameliorating their effect.

## Supporting Information Available

Mercury intrusion porosimetry results, Clipsham limestone; spectroscopic nomenclature; wavelength calibration; reproducibility kinetics; relationship between phase II diffusivity for HDO transition and % relative humidity (PDF)
