# Supplementary material for: Determining Water Transport Kinetics in Limestone by Dual-Wavelength Cavity Ring-Down Spectroscopy
Source: Anal Chem. 2022 Feb 8;94(7):3126–34. doi: 10.1021/acs.analchem.1c04277 (PMC9082503; doi:10.1021/acs.analchem.1c04277)
Supplement: Supplementary file 1 — ac1c04277_si_001.pdf [file ac1c04277_si_001.pdf]

Supplementary Information - Determining water transport kinetics in limestone by dual wavelength cavity ring-down spectroscopy

Dáire E. Browne,[†,‡] Robert Peverall,[†] Grant A. D. Ritchie,[*,†] and Heather A. Viles[‡]

†*Department of Chemistry, Physical and Theoretical Chemistry Laboratory, University of Oxford, South Parks Road, Oxford, OX1 3QZ, United Kingdom*

‡*School of Geography and the Environment, University of Oxford, South Parks Road, Oxford, OX1 3QY, United Kingdom*

* E-mail: grant.ritchie@chem.ox.ac.uk

Contents

1 Stone samples - Clipsham limestone **S2**

2 Spectroscopic nomenclature **S2**

3 Wavelength calibration **S3**

4 Reproducibility **S3**

5 Phase II diffusivity and % relative humidity **S4**

References **S6**

1 Stone samples - Clipsham limestone

This study investigated the use of CRDS to monitor moisture release using Clipsham limestone as the exemplar limestone. Mercury intrusion porosimetry[1] was carried out by the British Geological Survey to characterise the internal structure of the stone, and determine the open porosity of the sample. The pore size distribution is presented in figure S1 and shows that the Clipsham limestone used in this study has a unimodal pore distribution, with a median pore diameter of 1.18 μm. The Clipsham limestone samples have a density of 2.69 g mL^{-1} and an open porosity of 20.39 %.

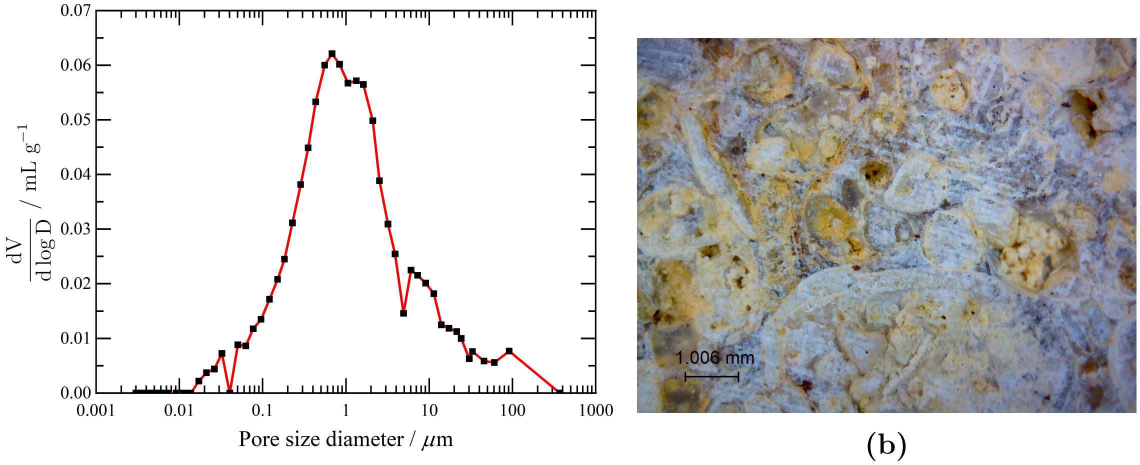

(a)

(b)

Figure S1: (a) Pore size distribution of Clipsham obtained by mercury intrusion porosimetry carried out by the British Geological Survey. **(b)** Micrograph of Clipsham limestone.

2 Spectroscopic nomenclature

Water is an asymmetric top molecule, with three unique moments of inertia. The rotational levels for water are reported in terms of J, the total angular momentum quantum number, K_a, the projection quantum number along the a axis, and K_c, the projection quantum number along the c axis. Water has three vibrational modes: ν_1 is the symmetric stretch, ν_2 is the symmetric bend and ν_3 is the asymmetric stretch. The ro-vibrational transitions

are reported by the angular momentum and vibrational quantum numbers of the lower and upper levels as the following:

$$J', K_a', K_c' \ (\nu_1' \ \nu_2' \ \nu_3') \ \leftarrow \ J'', K_a'', K_c'' \ (\nu_1'' \ \nu_2'' \ \nu_3'') \tag{1}$$

The transitions of interest in this work are the:

- H_2O 10,3,7 (021) ← 11,3,8 (000) transition at 6638.91 cm^{-1};
- H_2O 6,0,6(031) ← 7,0,7(010) transition at 6636.57 cm^{-1};
- HDO 8,6,2(210) ← 9,6,3(000) transition at 6638.17 cm^{-1}.

3 Wavelength calibration

The emission wavelength of the DFB laser is strongly dependent on its temperature and operating current, and in this experiment the fine tuning of the wavelength was made by controlling the laser temperature. Wavelength calibration has been carried out using a Burleigh WA-1000 wavemeter at constant laser drive current of 120 mA. This is shown in figure S2.

4 Reproducibility

To test the reproducibility of the CRDS measurement, and also variation between samples of the same limestone, the experiment of measuring α_{p} as a function of time with a 1.5 slm dry N_2 flow was repeated with four *ca.* $25 \times 25 \times 5$ mm Clipsham limestone samples. Figure S3 shows the normalised mass difference curves for the four samples and table S1 reports their drying kinetics. As expected there are slight variations due to differences between the samples but overall good agreement between the different parameters determined.

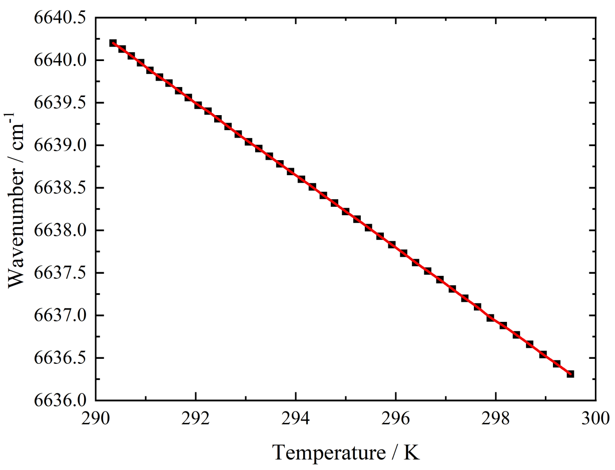

Figure S2: Wavelength calibration curve for the 1506 nm DFB laser as a function of temperature, measured at constant current of 120 mA. This was measured using the Burleigh WA-1000 wavemeter and fitted with a fourth order polynomial.

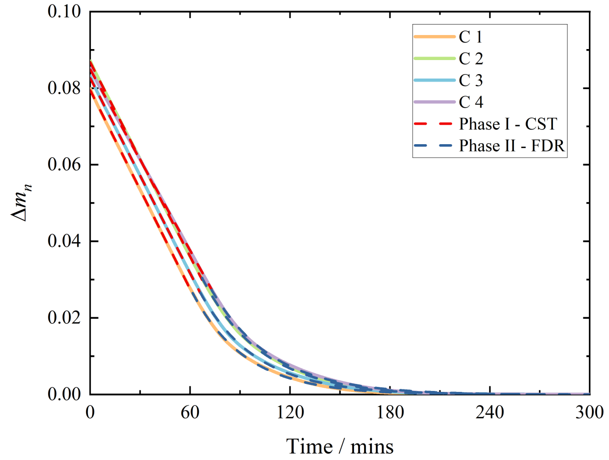

Figure S3: A plot of normalised mass difference as a function of time for four *ca.* $25 \times 25 \times 5$ mm samples of Clipsham limestone drying under a constant 1.5 slm N_2 flow.

5 Phase II diffusivity and % relative humidity

Phase II diffusivity is determined by unsaturated flow within the material[2] and D_{II} can therefore be determined from a linear relationship between the logarithmic terms and time:[3]

$$\ln\left(1 - \frac{m_{H_2O}(t)}{m_{H_2O}(\infty)}\right) - \ln\left(\frac{8}{\pi^2}\right) = -\frac{D_{II}\pi^2 t}{4L^2} \qquad (2)$$

Table S1: Comparison of drying kinetics using different samples of Clipsham limestone, all *ca.* 25 × 25 × 5 mm. Note: $m_{H_2O}(\infty)$ = spectroscopic mass; t_c = critical time; $\dot{m}_{CST}$ = constant mass flux for phase I; D_{II} = phase II diffusivity.

Sample	C1	C2	C3	C4
$m_{H_2O}(\infty)$ / g	0.361 ± 0.003	0.400 ± 0.003	0.393 ± 0.003	0.408 ± 0.003
Gravimetric mass / g	0.360 ± 0.005	0.390 ± 0.005	0.390 ± 0.005	0.410 ± 0.005
t_c / min	61.50	67.18	66.11	72.89
Critical saturation value / %	33	35	31	32
$\dot{m}_{CST}$ / kg m^{-2} s^{-1}	$1.13 \times 10^{-4} \pm 9 \times 10^{-6}$	$1.09 \times 10^{-4} \pm 9 \times 10^{-6}$	$1.16 \times 10^{-4} \pm 9 \times 10^{-6}$	$1.08 \times 10^{-4} \pm 9 \times 10^{-6}$
D_{II} / m^2 s^{-1}	$3.0 \times 10^{-9} \pm 1 \times 10^{-10}$	$2.8 \times 10^{-9} \pm 2 \times 10^{-10}$	$3.2 \times 10^{-9} \pm 4 \times 10^{-10}$	$3.0 \times 10^{-9} \pm 2 \times 10^{-10}$

Figure S4 shows D_{II} as a function of % relative humidity for the HDO 8,6,2(210) ← 9,6,3(000) transition at 6638.17 cm^{-1}, measured for two *ca.* 25 × 25 × 5 mm samples of Clipsham limestone. The results shown in figure S4 indicate that D_{II}(HDO) decreases with increasing humidity, implying that the external conditions still have an influence on the drying behaviour observed during phase II. Thus, this raises a question about the interpretation of the later drying times, especially with increasing % RH.

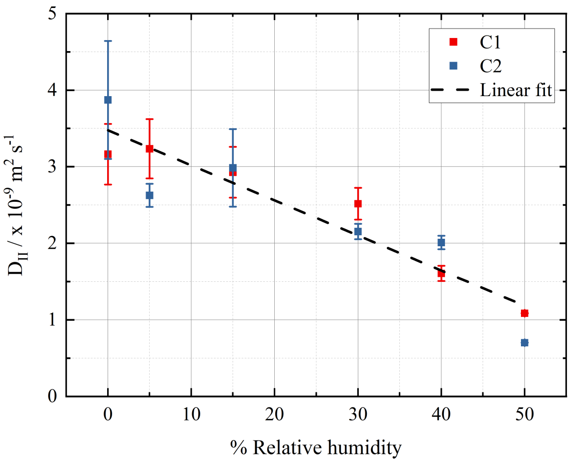

Figure S4: A plot of the phase II diffusivity, D_{II}, as a function of increasing humidity for two samples of *ca.* 25 × 25 × 5 mm Clipsham limestone samples.

References

(1) Giesche, H. Mercury Porosimetry: A General (Practical) Overview. *Part. Part. Syst. Charact.* **2006**, *23*, 9–19.

(2) Hall, C.; Hoff, W. D. *Water transport in brick, stone and concrete*, 2nd ed.; Spon Press, 2012; Chapter 7, pp 200–220.

(3) Crank, J. *The Mathematics of Diffusion*, 2nd ed.; Oxford University Press, 1975; Chapter 4, pp 44–68.
